# Do We Need to Detect Isoniazid Resistance in Addition to Rifampicin Resistance in Diagnostic Tests for Tuberculosis?

**DOI:** 10.1371/journal.pone.0084197

**Published:** 2014-01-03

**Authors:** Claudia M. Denkinger, Madhukar Pai, David W. Dowdy

**Affiliations:** 1 Department of Medicine, Beth Israel Deaconess Medical Center, Boston, Massachusetts, United States of America; 2 McGill International TB Centre & Department of Epidemiology, Biostatistics, and Occupational Health, McGill University, Montreal, Quebec, Canada; 3 Respiratory Epidemiology & Clinical Research Unit, Montreal Chest Institute, Montreal, Montreal, Quebec, Canada; 4 Department of Epidemiology, Bloomberg School of Public Health, Baltimore, Maryland, United States of America; 5 Center for Tuberculosis Research, Johns Hopkins University, School of Medicine, Baltimore, Maryland, United States of America; University of California Davis, United States of America

## Abstract

**Background:**

Multidrug-resistant tuberculosis (MDR-TB) is resistant to both rifampicin (RIF) and isoniazid (INH). Whereas many TB diagnostics detect RIF-resistance, few detect INH-monoresistance, which is common and may increase risk of acquired MDR-TB. Whether inclusion of INH-resistance in a first-line rapid test for TB would have an important impact on MDR-TB rates remains uncertain.

**Methods:**

We developed a transmission model to evaluate three tests in a population similar to that of India: a rapid molecular test for TB, the same test plus RIF-resistance detection (“TB+RIF”), and detection of RIF and INH-resistance (“TB+RIF/INH”). Our primary outcome was the prevalence of INH-resistant and MDR-TB at ten years.

**Results:**

Compared to the TB test alone and assuming treatment of all diagnosed MDR cases, the TB+RIF test reduced the prevalence of MDR-TB among all TB cases from 5.5% to 3.8% (30.6% reduction, 95% uncertainty range, UR: 17–54%). Despite using liberal assumptions about the impact of INH-monoresistance on treatment outcomes and MDR-TB acquisition, expansion from TB+RIF to TB+RIF/INH lowered this prevalence only from 3.8% to 3.6% further (4% reduction, 95% UR: 3–7%) and INH-monoresistant TB from 15.8% to 15.1% (4% reduction, 95% UR: (-8)-19%).

**Conclusion:**

When added to a rapid test for TB plus RIF-resistance, detection of INH-resistance has minimal impact on transmission of TB, MDR-TB, and INH-monoresistant TB.

## Introduction

Globally, tuberculosis (TB) occurs in about 9 million people, and kills about 1.4 million every year [1]. Although progress has been made in increasing TB cure rates, drug resistance is of increasing concern in most parts of the world. The current cornerstone of TB diagnosis (smear microscopy) does not detect drug resistance, but novel molecular tests offer the promise of rapidly detecting both TB and drug resistance simultaneously. For example, Xpert MTB/RIF (“Xpert”, Cepheid, Inc, Sunnyvale, CA, USA) [2], an automated molecular test, can detect active pulmonary TB and rifampicin (RIF) resistance without the need for a high-level lab infrastructure [3], [4]. RIF-resistance is a good surrogate marker for multidrug resistance (MDR) in high-burden settings and has therefore been prioritized by both Xpert and “fast-follower” tests (e.g. Genedrive, Epistem Ltd., Manchester, UK) [5], [6]. Therapy of MDR-TB guided by drug-susceptibility testing (DST) results in substantially improved treatment outcomes [7], [8], but the impact on population-level transmission is less clear [9].

Although the prevalence of isoniazid (INH) resistance is much higher than that of RIF [3], [10], detection of INH-resistance has received lower priority, largely because the clinical impact of INH-monoresistance is less pronounced. The extent of treatment failure, recurrence, and acquisition of further resistance development in patients with INH-monoresistance remains an issue of debate [11]–[15]; however, a recent meta-analysis suggests higher rates of failure or relapse and acquired resistance [12], [14]. Currently, the only World Health Organization (WHO)-endorsed assays capable of rapidly detecting INH-resistance (i.e. line-probe assays) require a lab with biosafety level 3 [16]. Whether inclusion of INH-resistance in a first-line rapid test for TB would have an important impact on MDR-TB rates remains uncertain. Thus, to address this question, we constructed a transmission model of a TB epidemic in a population patterned on that of India, an area with a high TB burden and growing concerns about emergence of drug resistance (MDR-prevalence 2.1% in new cases and 15% in retreatment cases in 2011) [17].

## Methods

### Model structure

**Figure 1:**
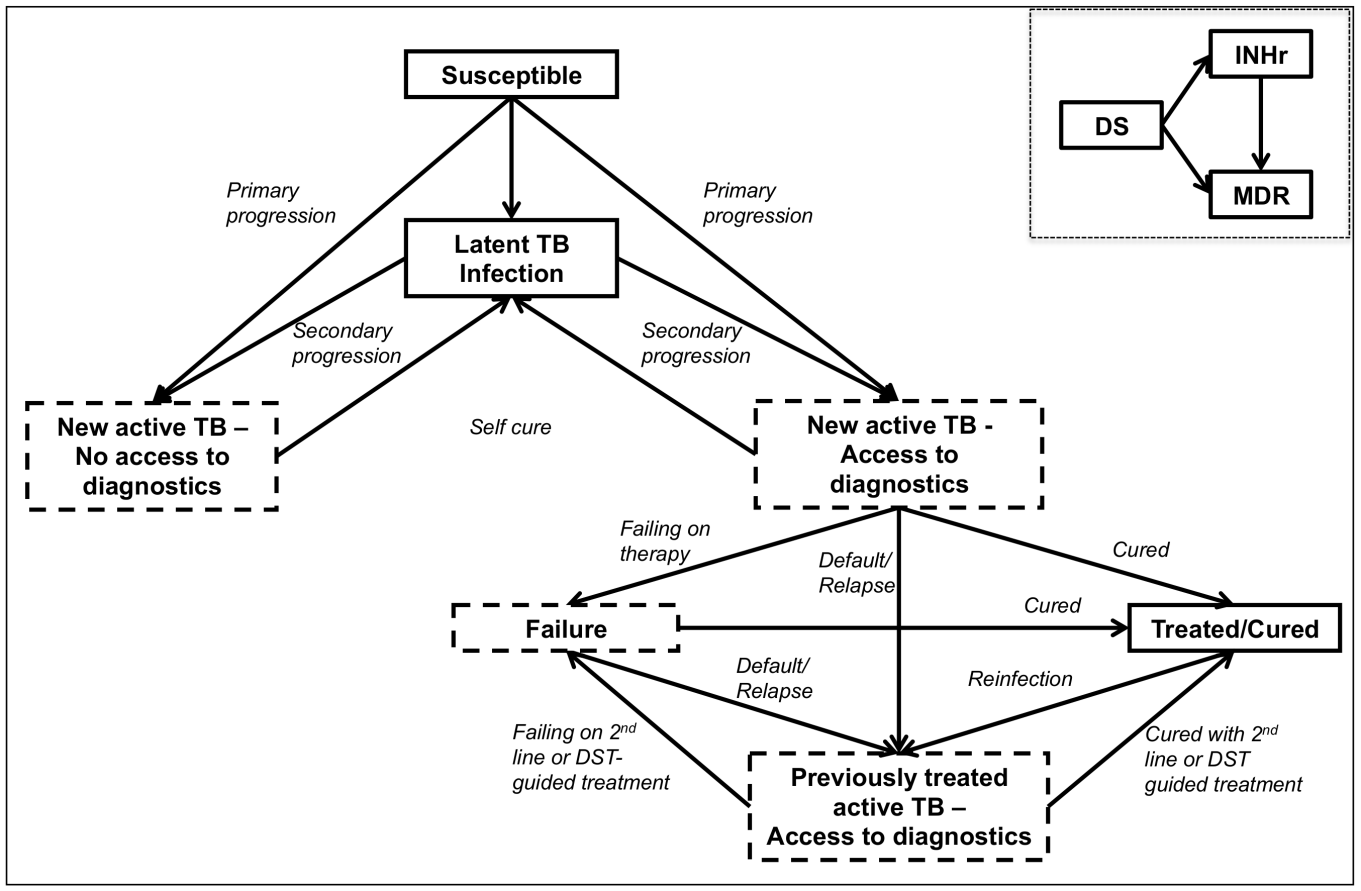
Study flow diagram. Dashed boxes contain subjects that are infectious. All latent active, failure and treated/cured boxes are subdivided by drug sensitivity (sensitive; multi-drug resistant, MDR; isoniazid monoresistant, INHr). As illustrated in the inset, MDR may arise directly from susceptible strains or (with increased probability) from INHr-TB.

We built a compartmental model using ordinary differential equations to describe a mature tuberculosis epidemic in a stable, homogeneously mixing population of adults aged 18 – 60 years with an incidence of TB and MDR-TB similar to that in India [18], [19]. Figure 1 describes the basic structure of the model; Table 1 lists the main parameters. A more detailed description is found in the online data supplement.

**Table 1:** Definition and values of key model parameters.

Definition		Value	Range	Reference
Birth/non-TB death rate per year		0.017	0.015–0.018	
TB mortality per year		0.15	0.10–0.22	[38]
Transmission events per infectious person-year) after year 0		6.77		[39]
Attenuation of infectiousness of INHr stains		0. 986	0.85–1.0	[30], [40], [41]
Attenuation of infectiousness of MDR strains		0.774	0.6–0.923*	[30], [42], [43]
Partial immunity afforded by previous infection		0.45	0.4–0.55	[44]–[46]
Proportion of TB infections progressing rapidly to active TB		0.14	0.05–0.14	[47]
Endogenous reactivation rate per year		0.0005	0.08–1.4 x10^−3^	[48]
Rate of self-cure in active TB per year		0.1	0.08–0.25	[19], [49]
Percent of patients without access to diagnostics		15	5–25	[50]
Sensitivity of current diagnostic standard		0.80	0.6–0.9	[38]
Sensitivity of molecular methods		0.95	0.75–0.98	[51], [52]
Proportion of patients initiating treatment after diagnosis		0.85	0.81–0.89	[53], [54]
Treatment outcomes of patients with DS-TB+				[38], [55]–[57]
	Cured	0.88	0.75–0.95	
	Developing INHr-TB	0.004	0.003–0.01	
	Developing MDR-TB	0.001	0.0005–0.005	
Treatment outcomes of patients with INHr -TB on standard therapy+				[7], [12], [38], [56]–[59]
	Cured	0.80	0.65–0.90	
	Developing MDR-TB	0.01	0.001–0.02	
Treatment outcomes of patients with INHr -TB on DST-guided therapy+				[10], [30], [38], [46]–[49]
	Cured	0.88	0.75–0.95	
	Developing MDR-TB	0.001	0.001–0.005	

upper margin defined by value necessary to attain doubling of proportion MDR over the duration of model period

Further information on treatment outcomes is available in Table S2 (in File S1) in the supplement; outcomes are expressed as probabilities per treatment episode.

Abbreviations: TB =  tuberculosis, INHr =  Isoniazid monoresistant, MDR =  multi-drug resistant (i.e., resistant to isoniazid and rifampin, DST =  drug-susceptibility testing, DS-TB =  drug-susceptible TB

The model population was divided into compartments defined by the individual's status of TB infection or disease and by the TB drug susceptibility pattern (sensitive, INH-monoresistant and MDR). As our goal was not to evaluate the role of INH testing on treatment of RIF-monoresistant TB, we assumed that all RIF-resistant TB (whether MDR-TB or only RIF monoresistant) would be treated equivalently [5].

### Input parameters

We used data from the WHO surveillance systems and other published literature to inform our model (Table 1 as well as Table S1 and S2 in File S1 in the online data supplement) [1], [10], [20].

### Calibration of model

We first established a baseline “year zero,” modeled as a scenario representative of the current TB epidemic (including INH-monoresistant and MDR-TB) in India [1]. We initiated the model at steady state 60 years prior to year zero (e.g., 1951, if year zero corresponds to 2011), calibrating the TB transmission rate (number of secondary infections per smear-positive person-year) to match India's WHO-estimated 2011 TB incidence (181 per 100,000/year) [1]. From this equilibrium, we initially planned to allow INH-monoresistant and MDR-TB to emerge at a constant rate over 60 years. However, the assumption of constant emergence of resistance over 60 years leading to present levels of resistance required epidemiologically implausible assumptions such as higher transmission fitness of INH-monoresistant TB relative to wild-type or treatment success for INH-monoresistant TB below 70%.

Thus, we instead calibrated the transmission rate of INH-monoresistant TB to provide a steady-state level of INH-monoresistance (at 15% of new cases) over the past 60 years. This is consistent with data of high INH-monoresistance from early surveillance reports and the lack of a significant increase in INH-resistance in India since that time [1], [10], [21], [22]. This procedure required only a minimal decrease in the transmission fitness of INH-monoresistant. After initiating this steady state, we calibrated the relative infectiousness of MDR-TB such that the modeled incidence of MDR-TB among new (not previously treated) cases was 2.1%, as estimated in India in 2011 [1]. In year zero we also reduced the overall TB transmission rate to a degree sufficient to generate a 2% per year decline in TB incidence, the globally-estimated average [1].

### Baseline

At baseline (year zero), we assumed a “standard” diagnostic approach for individuals suspected of having pulmonary TB; this approach may consist of multiple sputum smear examinations, ancillary diagnostic tests (e.g. chest X-ray), and clinical judgment. We calibrated the sensitivity of this “standard approach” to a value that provided a reasonable estimate of TB case detection (model value 75%, RNTCP estimate for smear-positive cases 70%) [20]. We assumed that this “standard approach” resulted in diagnosis and treatment of MDR only among patients who have failed initial therapy.

### Different scenarios considered

Starting in year zero, we augmented the “standard” diagnostic approach with a molecular diagnostic test for TB, but with no capacity to detect drug resistance. We assumed that the diagnostic test would increase the sensitivity of the standard approach from 75% to 95% (i.e., detecting 80% of TB cases who would otherwise be missed) (6). We assumed the same baseline level of molecular testing in all scenarios. Specifically, we considered that, beginning in year zero (the year in which the test was rolled out), 50%, 80%, and 100% of new, previously treated, and failure cases respectively would receive this testing, among those patients who had any access to appropriate diagnosis. We assumed that 85% of patients that were diagnosed were going to receive treatment [6], [23], [24]. In a sensitivity analysis, we compared this to a “low-coverage” scenario in which only 15%, 25%, and 30% of patients had access to the test.

For each level of coverage, we compared the molecular TB detection scenario against a series of alternative scenarios in which the novel molecular test was assumed also to have the ability to detect resistance to RIF with sensitivity of 94% [25] (TB+RIF; e.g. current version of Xpert), or RIF with sensitivity of 94% and isoniazid with sensitivity of 88% (TB+RIF/INH, i.e., complete detection of the two most common resistance mutations, katG and inhA, which account for 88% of all INH-resistant cases [26]).

### Aims and outcomes

Our primary modeling aim was to assess the maximum potential impact of adding a test for INH-resistance to a molecular test with capacity to detect resistance to RIF. As such, we made liberal assumptions about the impact of INH-monoresistance, including a reduction in the proportion cured from 88% (drug-susceptible) to 80% (INH-monoresistant) and a ten-fold increase in probability of acquiring MDR-TB under standard therapy (from 0.1% to 1%), as well as immediate implementation and ability to improve treatment outcomes (e.g., by use of a quinolone) when INH-monoresistance was detected [12], [27]. To the extent that these assumptions may overestimate the clinical importance of INH-monoresistance, the impact of detecting such resistance will likewise be overestimated. We also assumed that detection of MDR-TB universally leads to appropriate second-line therapy (Table S2 in File S1).

Our primary outcomes were the projected prevalence of INH-monoresistant TB and MDR-TB in each scenario and, secondarily, incidence and mortality due to TB. For drug-resistant strains, we report outcomes both as a proportion and as an absolute number of detected cases (regardless of whether cases are appropriately diagnosed as drug resistant). This latter outcome corresponds to what might be seen in an idealized population-level surveillance system capable of detecting drug susceptibility among all diagnosed cases.

### Sensitivity analysis

We conducted a sensitivity analyses on all model parameters with one-way variation in a parameter value (i.e., holding other parameter values constant), taking as the outcomes the difference in MDR-TB as a proportion of detected cases comparing TB+RIF/INH to TB+RIF detection alone. The ranges of the parameters are based on the available literature and possible advances in the near future (e.g. reaching 100% sensitivity in INH-resistance detection), as outlined in Table S4 in File S1.

To estimate variability associated with simultaneous changes in all parameters, we also conducted a probabilistic uncertainty analysis, using Latin Hypercube Sampling (additional detail on the method is provided in an online data supplement).

## Results

In the absence of any improvement in TB diagnosis, we projected that MDR-TB would gradually rise as a percentage of cases from 2.1% among incident and 5.0% of all cases in year zero to 2.4% among incident and 5.7% among all cases by year ten. If a molecular test for TB – without RIF detection – were implemented at year zero (50%, 80%, 100% coverage among new, previously treated and failure cases, respectively, excluding those with no access to care), the proportion of TB cases with MDR increase more slowly (2.2% of incident cases, 5.5% of all cases). The ability of a novel test to reduce the increase in MDR-TB was a function of population coverage; if coverage of the novel test were lower (15%, 25%, and 30% among new, previously treated and failure), MDR was responsible for 2.3% of incident TB and 5.6% of all TB by year ten (Table 2).

**Table 2:** Projected tuberculosis outcomes.

		10-year projected TB outcomes							
	Incidence (per 100,000)	Mortality (per 100,000)	MDR among new cases (%)		MDR among retreat-ment cases (%)	MDR among total cases (%)	INH among new cases (%)	INH among retreat-ment cases (%)	INH among total cases (%)
Low coverage scenario									
TB detection only	139.4	26.9	2.2		18.7	5.6	14.8	18.7	15.6
TB + RIF	139.1	26.8	2.1		16.4	4.9	14.8	19.2	15.7
TB + RIF/INH	139.0	26.8	2.1		16.3	4.9	14.8	18.3	15.5
High coverage scenario									
TB detection only	128.0	24.4	2.2	18.9		5.5	14.8	18.2	15.5
TB + RIF	127.3	24.1	1.7	13.0		3.8	14.9	19.5	15.8
TB + RIF/INH	127.1	24.1	1.7	12.5		3.6	14.8	16.8	15.2

Incidence, mortality and proportion of resistant among detected new, relapse/default and total cases by year ten with different interventions in high- and low-coverage scenarios.

**Figure 2:**
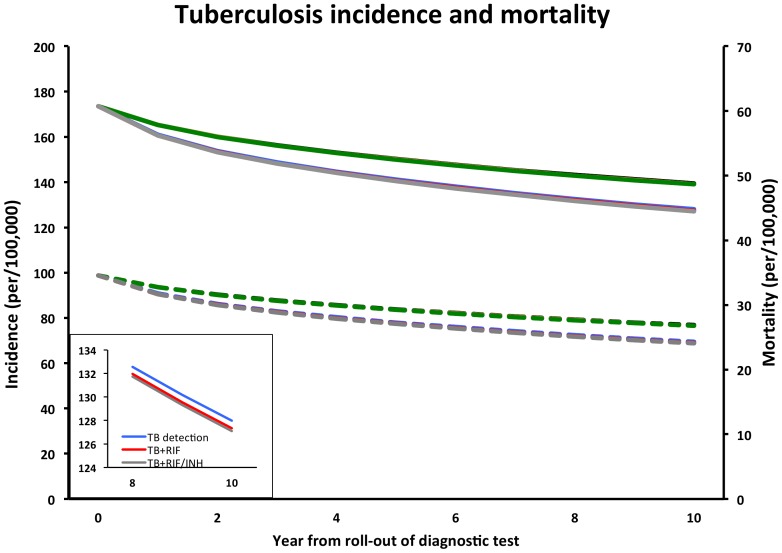
Impact of resistance testing on incidence and mortality. Trajectory of overall TB incidence (solid lines, left axis) and mortality (dotted lines, right axis) over 10 years with introduction of a molecular test for diagnosis and detection of rifampin (RIF) resistance, with or without a molecular test for isoniazid (INH) resistance. Grey lines correspond to the high-coverage scenario (i.e. 50%, 80% and 100% coverage among new, previously treated and failure cases, respectively, excluding those with no access to care), green lines to an alternative lower-coverage scenario (15%, 25%, and 30% among new, previously treated and failure). The curves for TB+RIF versus TB+RIF/INH are indistinguishable on the graph because the projected outcomes are so similar (see inset with incidence from year 8–10 for high coverage scenarios).

We then evaluated the impact of adding RIF detection to the novel molecular test. In the high-coverage scenarios, the addition of RIF-resistance testing to a molecular test alone caused MDR-TB overall prevalence to fall from 5.5% to 3.8% (a relative reduction of 30.6% in year 10 (95% UR: 17–54%). However, the impact on the overall burden of TB incidence (reduction of 0.6 per 100,000/year, 95% UR: 0.2–2.2) and mortality (reduction of 0.2 per 100,000/year, 95% UR: 0.07–0.5) was minimal. The effect in low-coverage scenarios was accordingly smaller (Figure 2 and Table 2).

**Figure 3:**
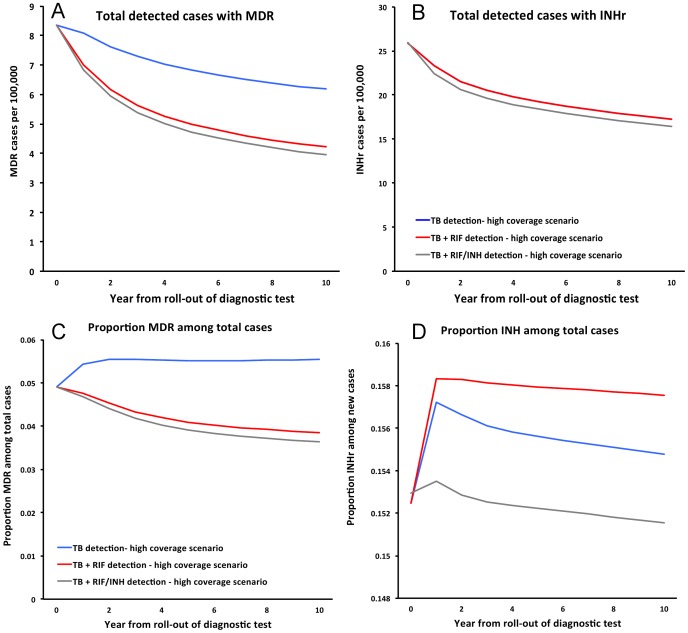
Impact of resistance testing on multi-drug and isoniazid resistance. Projected trajectories for multi-drug resistant (MDR) (A, C) and INH-resistance (INHr) (B, D) cases with TB detection, TB+RIF and TB+RIF/INH over ten years. Results are shown as the absolute number of MDR or INHr cases per 100,000 (A, B) and as a proportion of all cases (new cases and relapse/default cases) detected (C, D).

Finally, we compared the incremental benefit of adding INH resistance testing to the TB+RIF test. Figure 2 presents the TB incidence and TB mortality on a year-by-year basis over 10 years comparing different scenarios of coverage and testing for drug susceptibility; the projected trajectories for TB+RIF versus TB+RIF/INH in this Figure are so similar as to be visually indiscernible. Figure 3 shows corresponding trends in MDR-TB and INH-monoresistant TB cases in the high coverage setting as the absolute number of cases detected per 100,000 (A, B) and as a proportion of all cases (new cases and relapse/default cases) detected (C, D). Again here, while addition of RIF detection caused a substantial decline in MDR-TB proportion (i.e., 30.6% over 10 years), the further addition of detecting INH resistance generated few additional gains.

Despite the sizeable impact of RIF-resistance testing on MDR-TB (5.5% to 3.8% with a relative reduction of 30.6%) as well as very liberal assumptions about the impact of and the ability to treat INH-monoresistance (e.g., ten-fold risk of acquiring MDR-TB and improved probability of treatment success if INH-monoresistant TB detected), the addition of INH-resistance testing had virtually no detectable incremental impact on MDR-TB prevalence relative to detection of TB+RIF. Adding INH resistance detection resulted in a projected 4% reduction (95% UR: 3–7%) in the proportion of total cases that are MDR-TB over 10 years, from 3.8% to 3.6% (Table 2; Figures 3A and 3C, red versus grey lines), with the upper bound of the 95% UR corresponding to a 7% reduction (from 3.8% to 3.5%) and the lower bound resulting in <1% reduction. Effects on INH-monoresistant TB were similarly small (4% reduction from 15.8% to 15.1% compared to TB+RIF scenario, 95% UR: (-8)-19%; Table 2, Figure 3B and D). The number of INH-resistant TB among all detected cases was unchanged in a TB+RIF scenario and decreased by only 0.8 per 100,000 (4.8% relative reduction) in a TB+RIF/INH scenario (Figure 3D).

The projected incremental impact of testing for TB+RIF/INH was not qualitatively changed in scenarios that considered INH-monoresistant TB to be equally transmissible to wild-type, under-reporting INH-monoresistant TB to the WHO by a factor of two, or doubling of MDR-TB prevalence over ten years (e.g., through compensatory mutation) (see online data supplement).

**Figure 4:**
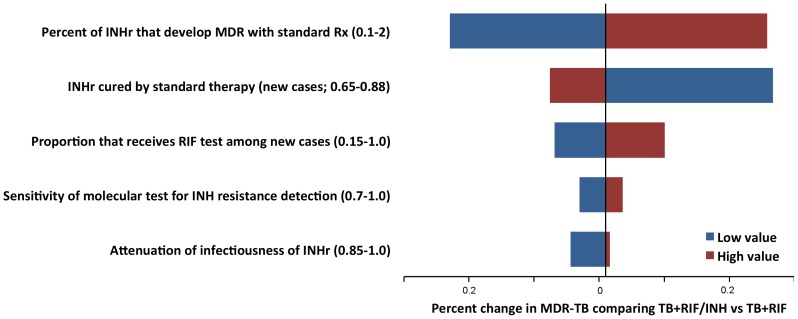
Sensitivity analysis. Absolute percent change of MDR among all cases by year 10 if scenarios with TB+RIF and TB+RIF/INH are compared and one variable (in y-axis) is changed over the range defined in Table S4 in File S1.

On sensitivity analysis, the per cent of patients who develop MDR and the proportion on INH-resistant patients that is cured if an INH-resistant strain is treated with standard therapy affect the incremental benefit of INH resistance testing the most. However, the benefit is small and no parameter, if varied in one direction within the ranges defined in Table S4 in File S1 (while others held constant) changes the absolute per cent of MDR among all cases by more than 0.3% in year 10 if a scenario with TB+RIF and a scenario with TB+RIF/INH are compared (Figure 4). Further supplementary analyses are provided in the supplement (Figure S1, S2, S3, S4, S5, S6).

## Discussion

This transmission model of a TB epidemic in a population patterned on that of India suggests that, while detection of resistance to RIF (if followed by appropriate treatment) can reduce MDR-TB rates substantially and rapidly (30.6% reduction [95%UR: 17–54%] within ten years of implementation), the incremental impact of adding susceptibility testing for INH is likely to be very small. Specifically, a test for INH and RIF resistance – compared against testing for RIF-resistance alone – would only reduce INH-monoresistant TB and MDR-TB by a further 4% (for example, from 3.8% to 3.6%), despite very liberal assumptions about the epidemiological importance of INH-monoresistant TB and the ability to scale-up such a test. These results suggest that, while testing for INH monoresistance may still confer individual-level benefit (through improved outcomes if INH-resistance was recognized and the treatment adjusted accordingly) and also likely will be cost-effective, efforts to change population-level epidemiology of MDR-TB should focus on widely implementing rapid tests for TB and RIF-resistance rather than additionally incorporating tests for INH-monoresistance [28].

Though our focus was on the incremental effect of testing for INH-resistance, our model projected that implementation of the TB+RIF test had a substantial and rapid effect on MDR-TB rates. This projection reflects, in large part, more rapid diagnosis and cure of a large pool of prevalent (i.e. chronic) MDR cases. Our model projected that, at baseline, over 60% of MDR-TB transmission occurred after the first diagnostic attempt. Therefore, achieving a diagnosis of MDR-TB at initial presentation can result in a substantial reduction of MDR transmission. However, these projections assume high population coverage and universal treatment for MDR-TB upon diagnosis and thus likely overestimate the impact of such a test on MDR-TB rates as could be implemented in real-world settings [9], [20], [29].

Despite finding fairly dramatic effects for testing of RIF resistance on MDR-TB (30.6% decline, Figure 3), our model nonetheless suggests that the incremental population-level impact of an INH-resistance test will be limited. This finding may at least be partially due to the stability of INH-resistance rates over time, assumptions about transmission and competition between INH-susceptible and INH-resistant strains, and the relative lack of selective pressure for INH-monoresistance to emerge (in that INH-monoresistant strains remain largely curable, even with existing first-line therapy) [1], [12], [30]. Regarding the underlying dynamics of INH-monoresistant TB, our model demonstrates that, even assuming equivalent transmissibility of INH-monoresistant TB and drug-susceptible TB, levels of INH-monoresistance that are stable at 15–20% among new cases are realistic. Furthermore, unlike MDR-TB, scenarios that assume a gradual 60-year increase in rates of INH-monoresistant TB are largely untenable; offering indirect evidence that INH-monoresistance today largely reflects patterns that developed decades ago.

Our model, as with any mathematical representation, has certain limitations. In order to increase transparency and generalizability, the model uses a hypothetical population and is only calibrated to key input parameters (TB, INH and MDR incidence) reflective of the current TB epidemiology in India. This model does not, therefore, account for the complexity of the epidemiological scenario in India or any other single specific location [1], [20], [29], [31]. The simplified model structure also cannot fully capture the heterogeneity of TB epidemics, particularly those driven by MDR-TB, that could be further potentiated by heterogeneous mixing in high risk groups (e.g. in patients with HIV or in prisoners in countries of the former Soviet Union) or by a dysfunctional healthcare system [32]–[34]. For example, our model does not account for HIV as a driver of TB and TB drug resistance, which may be appropriate in settings like India but limits the generalizability of this model to settings such as Sub-Saharan Africa [1], [35].

Our model also does not take INH preventive therapy as a driver of INH-resistance into account. Uptake of IPT is very limited in India and similar settings [20], and an amplifying effect of IPT on emergence of INH-monoresistance, while possible, has not been convincingly demonstrated [36], [37].

Finally, by excluding RIF monoresistance from the model, we do not account for the potential cost savings associated with identifying such strains and foregoing MDR-TB treatment in such cases; high rates of RIF-monoresistance are primarily observed in countries with low overall MDR prevalence [5], and we may therefore underestimate the benefit of INH-monoresistance testing in those settings.

In conclusion, this model projects that addition of an INH-resistance test to an existing molecular test for TB+RIF will have minimal population-level effects on the prevalence of MDR or INH-resistance if scaled up in a population resembling India over a ten-year time span. In contrast, we project a sizable impact of molecular testing for TB+RIF if cases diagnosed with MDR are also given access to treatment. Efforts at improving diagnostic testing for TB and TB drug susceptibility should therefore prioritize more wide distribution of rapid testing for TB (including RIF-resistance) over deployment of additional tests to detect INH-resistance in individuals with active TB.

## Supporting Information

Figure S1 **Impact of resistance testing with a hypothetical increase of INH.** Assuming a 25% increase of INH cases among all cases detected in 2020 (through a compensatory mutation with equal transmissibility compared to sensitive cases and by presuming a cure rate with standard therapy of only 40%), the impact of a TB+RIF on the number of MDR cases among total cases detected and the proportion of MDR would be similar (A and C) compared to data shown in Figure 3, however the effect of a test for INH resistance would be somewhat enhanced. The proportion INH monoresistant at year 10 would be reduced from 23.7% to 18.5% in the TB+RIF to the TB+RIF/INH scenario (D), with a reduction of INH resistant cases by 8/100,000 (B). The proportion MDR would be 0.7% lower (C), with a total reduction of 1 MDR case per 100,000 (A). Note that in panel B the lines for scenarios with a molecular test only and a test that detects rifampin resistance are overlapping.
(TIFF)

Figure S2 **Impact of resistance testing with a hypothetical increase of MDR.** Assuming a doubling of multi-drug resistant (MDR) cases among all cases detected by year 10 (through a compensatory mutation that substantially increases transmissibility of MDR cases), the impact of a test for rifampin resistance would be more substantial (A and C) compared to a test for TB detection only; however, the additional effect of an INH resistance test on INH and MDR resistance would remain small. The proportion INH resistant cases at year 10 would be further reduced by 4.7% compared to the TB+RIF scenario (D), with a reduction of INH resistant cases by 6.5/100,000 in year ten (B). The proportion MDR would be decreased by 0.6% over a TB+RIF scenario (C), with a reduction in MDR cases of 1.6 per 100,000 (D). Note that in panel B the lines for the two scenarios TB detection only and TB+RIF are overlapping.
(TIFF)

Figure S3 **Impact of resistance testing with a higher INH prevalence.** Assuming that isoniazid (INH) resistance is underreported and it is in fact double as common as reported in year zero (30% instead 15% in new cases), the addition of a test for INH resistance still would have only a small effect on reducing the proportion of INH resistant cases and multi-drug resistant cases beyond that of a molecular test with rifampin resistance testing alone (A and B). The proportional impact of a TB+RIF scenario (C and D) would be similar to the scenario reported in 3C and 3D.
(TIFF)

Figure S4 **Impact of resistance testing on incidence and mortality over 50 years.** Trajectory of overall TB incidence (solid lines, left axis) and mortality (dotted lines, right axis) over 50 years with introduction of a molecular test for diagnosis and detection of rifampin (RIF) resistance, with or without a molecular test for isoniazid (INH) resistance. Grey lines correspond to the high-coverage scenario (i.e. 50%, 80% and 100% coverage among new, previously treated and failure cases, respectively, excluding those with no access to care), green lines to an alternative lower-coverage scenario (15%, 25%, and 30% among new, previously treated and failure). The curves for TB+RIF versus TB+RIF/INH are indistinguishable on the graph because the projected outcomes of incidence and mortality are so similar.
(TIF)

Figure S5 **Impact of resistance testing on multi-drug and isoniazid resistance over 50 years.** Projected trajectories for multi-drug resistant (MDR) (A, C) and INH-resistance (INHr) (B, D) cases with TB detection, TB+RIF and TB+RIF/INH over 50 years. Results are shown as the absolute number of MDR or INHr cases per 100,000 (A, B) and as a proportion of all cases (new cases and relapse/default cases) detected (C, D).
(TIF)

Figure S6 **Impact of resistance testing on multi-drug and isoniazid resistance over 10 years under different assumptions relating to superinfection.** Projected trajectories for multi-drug resistant (MDR) (A) and INH-resistance (INHr) (B) cases with TB detection, TB+RIF and TB+RIF/INH over 10 years. Results are shown as the absolute number of MDR or INHr cases per 100,000 for the high-coverage scenario with complete protection against superinfection versus no protection at all (i.e. superinfecting strain becomes the dominant strain).
(TIF)

File S1 **Includes Tables S1-S4.**
(DOC)
