# Supplementary figures and images for: Do We Need to Detect Isoniazid Resistance in Addition to Rifampicin Resistance in Diagnostic Tests for Tuberculosis?
Source: PLoS One. 2014 Jan 3;9(1):e84197. doi: 10.1371/journal.pone.0084197 (PMC3880287; doi:10.1371/journal.pone.0084197)

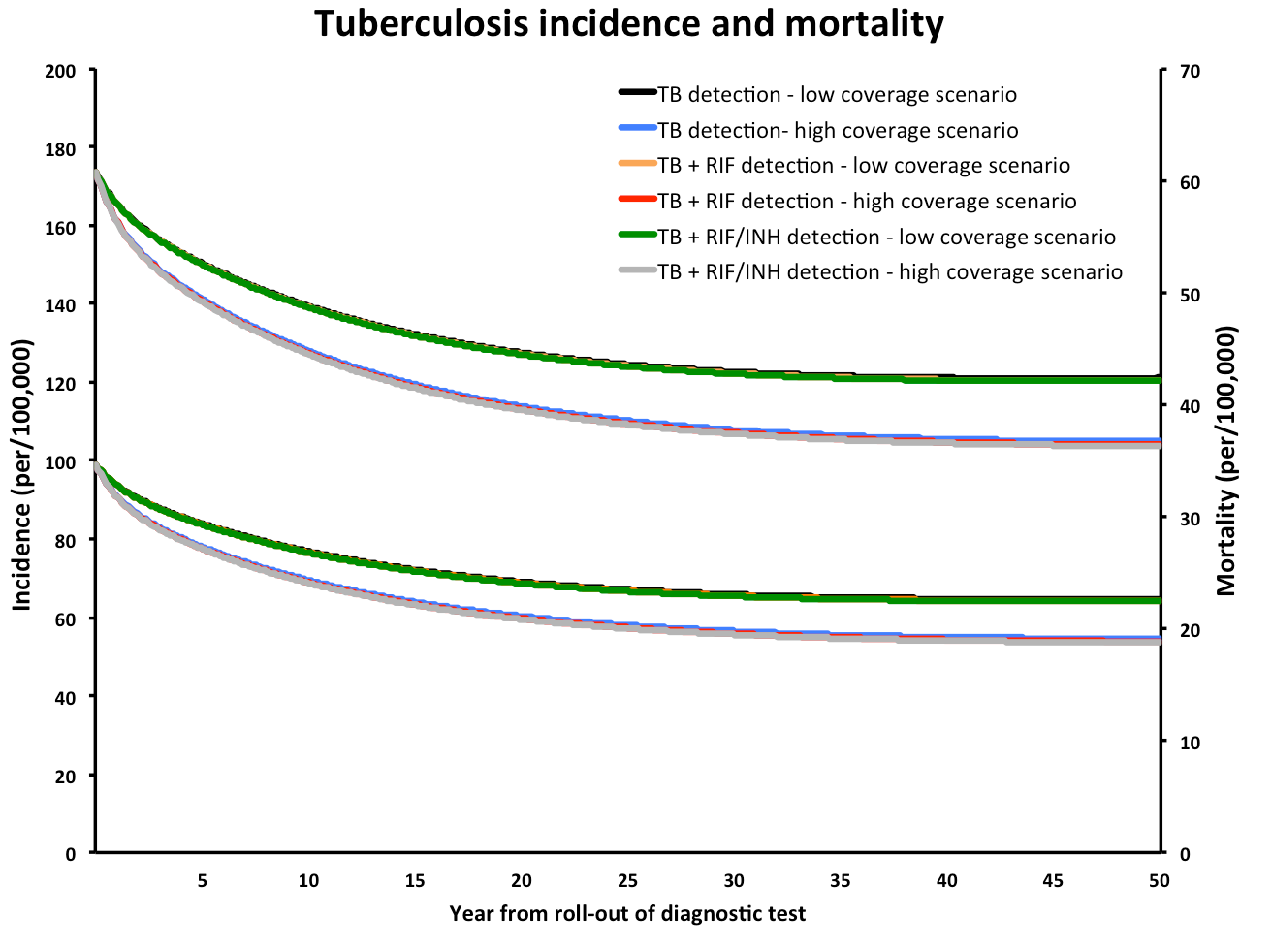

Supplement: Figure S4 — Impact of resistance testing on incidence and mortality over 50 years. Trajectory of overall TB incidence (solid lines, left axis) and mortality (dotted lines, right axis) over 50 years with introduction of a molecular test for diagnosis and detection of rifampin (RIF) resistance, with or without a molecular test for isoniazid (INH) resistance. Grey lines correspond to the high-coverage scenario (i.e. 50%, 80% and 100% coverage among new, previously treated and failure cases, respectively, excluding those with no access to care), green lines to an alternative lower-coverage scenario (15%, 25%, and 30% among new, previously treated and failure). The curves for TB+RIF versus TB+RIF/INH are indistinguishable on the graph because the projected outcomes of incidence and mortality are so similar. (TIF) [file pone.0084197.s004.tif]

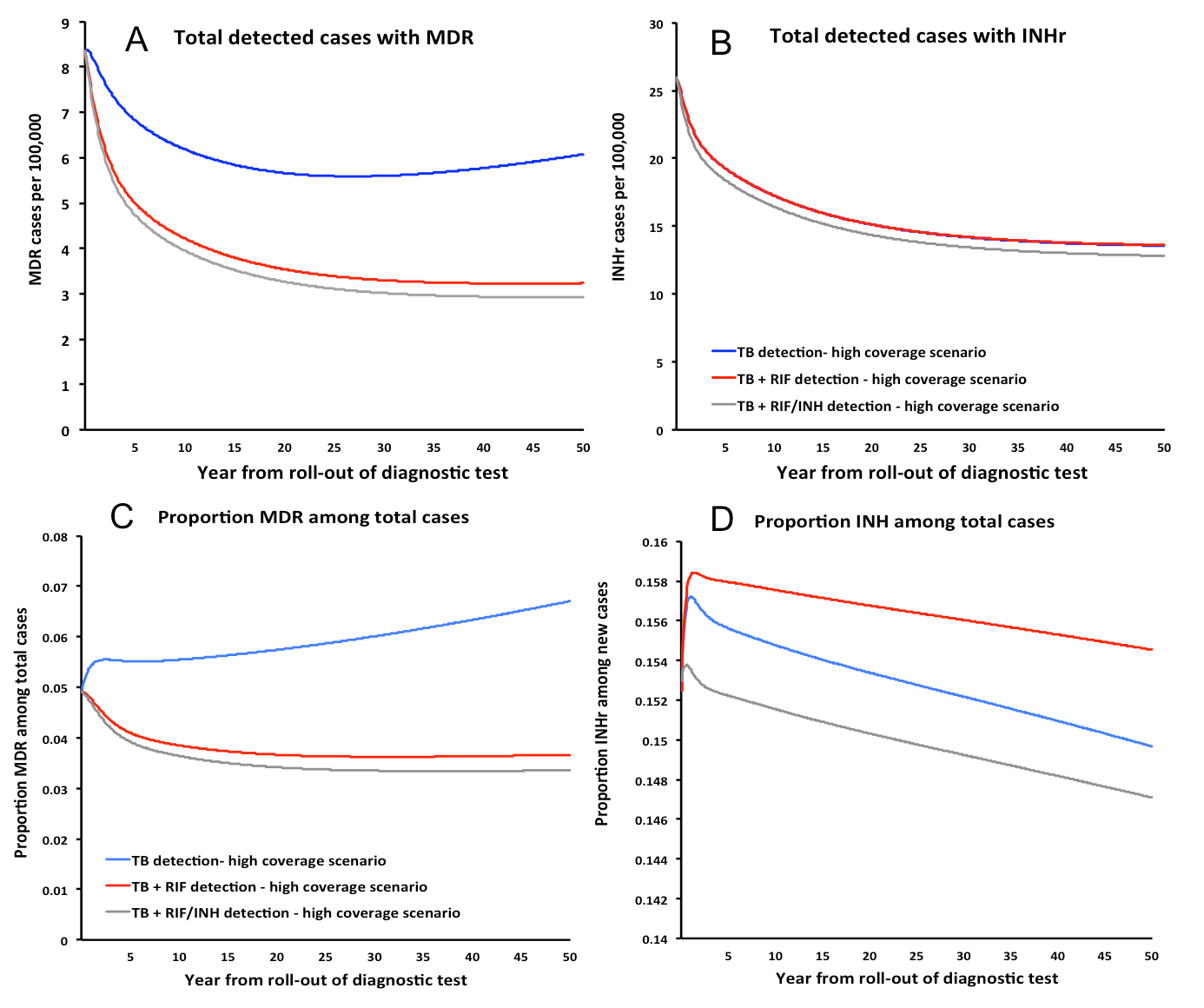

Supplement: Figure S5 — Impact of resistance testing on multi-drug and isoniazid resistance over 50 years. Projected trajectories for multi-drug resistant (MDR) (A, C) and INH-resistance (INHr) (B, D) cases with TB detection, TB+RIF and TB+RIF/INH over 50 years. Results are shown as the absolute number of MDR or INHr cases per 100,000 (A, B) and as a proportion of all cases (new cases and relapse/default cases) detected (C, D). (TIF) [file pone.0084197.s005.tif]

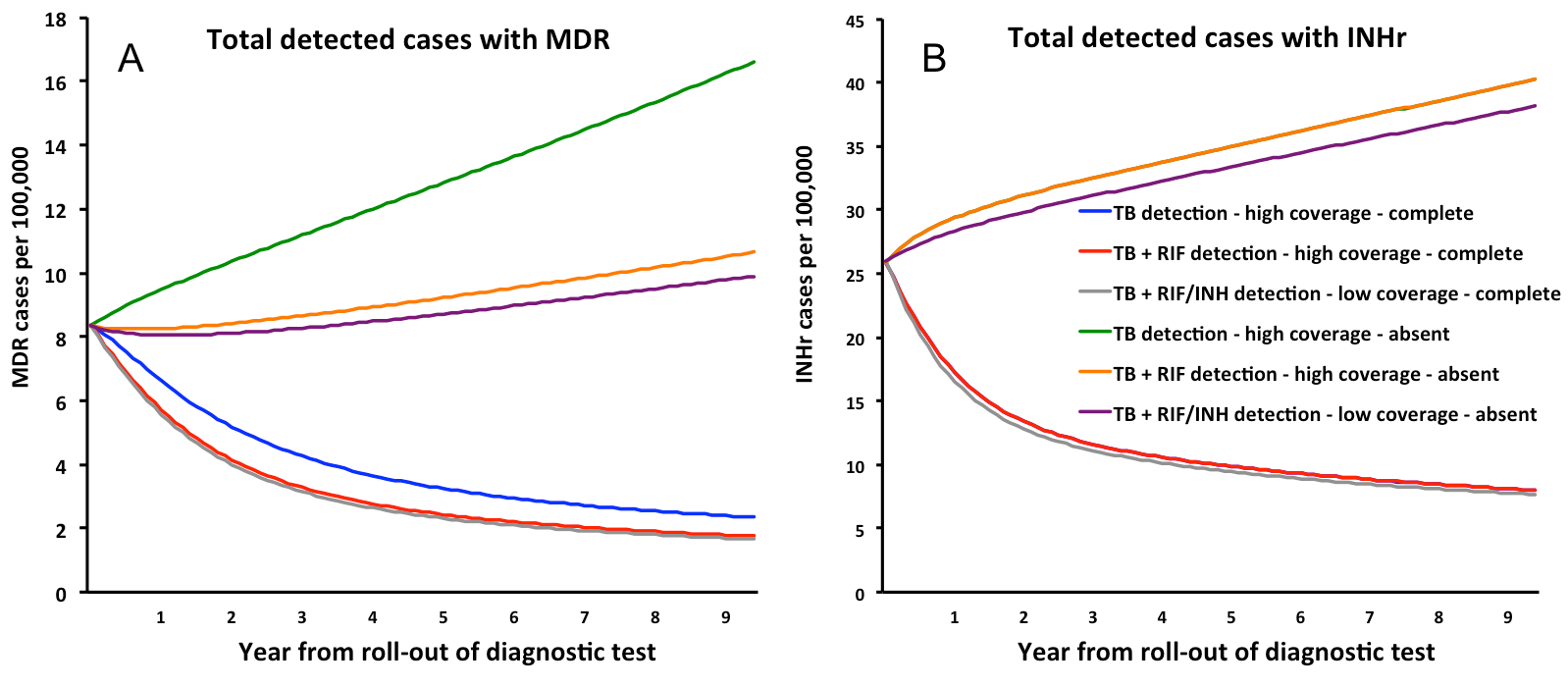

Supplement: Figure S6 — Impact of resistance testing on multi-drug and isoniazid resistance over 10 years under different assumptions relating to superinfection. Projected trajectories for multi-drug resistant (MDR) (A) and INH-resistance (INHr) (B) cases with TB detection, TB+RIF and TB+RIF/INH over 10 years. Results are shown as the absolute number of MDR or INHr cases per 100,000 for the high-coverage scenario with complete protection against superinfection versus no protection at all (i.e. superinfecting strain becomes the dominant strain). (TIF) [file pone.0084197.s006.tif]
