# Supplementary material for: Do We Need to Detect Isoniazid Resistance in Addition to Rifampicin Resistance in Diagnostic Tests for Tuberculosis?
Source: PLoS One. 2014 Jan 3;9(1):e84197. doi: 10.1371/journal.pone.0084197 (PMC3880287; doi:10.1371/journal.pone.0084197)
Supplement: File S1 — Includes Tables S1-S4. (DOC) [file pone.0084197.s007.doc]

**Do we need to detect isoniazid resistance in addition to rifampin resistance in the next generation of molecular diagnostics for tuberculosis?**

Claudia M. Denkinger, Madhukar Pai, David W. Dowdy

**Supplementary information**

1. **Model Structure Description**

We constructed compartmental difference-equation model to describe a mature tuberculosis (TB) epidemic in a stable population of 100,000 adults aged 18 – 60 years patterned on that of India. The model population is divided into compartments defined by the individual’s status of TB infection or disease and by the TB drug susceptibility pattern (nonresistant, multi-drug resistant (MDR), or isoniazid (INH)-monoresistant).

All individuals at any stage of TB infection are presumed to harbor a ‘dominant’ TB strain; this strain determines the patient’s drug-susceptibility pattern upon development of active TB. An individual’s risk of becoming infected with a specified TB strain (defined by drug resistance: sensitive, MDR, or INH-monoresistant) is directly proportional to the number of active TB patients harboring the specified strain at a given time, and the relative infectivity of that strain. Upon infection, the infecting strain will become the dominant strain in 100% of previously uninfected individuals, and a smaller proportion of individuals harboring latent TB infection because latent infection provides partial protection against reinfection (Table S1).

Among individuals in whom the infecting strain becomes the dominant strain, a proportion will progress rapidly to active TB, and the remainder will become latently infected with the new strain . Latently infected individuals remain at risk of endogenous reactivation with the same or reinfection with any other strain throughout their lifetimes (taking into account partial protection through prior infection) . Treatment for latent TB infection with INH is not incorporated into the model.

Upon development of active TB, patients are immediately considered to be infectious and have an increased mortality risk due to TB. Some patients will not have access to health care and diagnostics for TB and will remain infectious until they either self-cure or die (Table S1). Other patients will get diagnosed and treated. These patients will exit the subpopulation of active diagnosed cases at a rate defined as the inverse of the mean time to initial diagnosis (see diagnostic rate below).

The likelihood of being diagnosed and the time to initial diagnosis will depend on the diagnostic method available. Individuals with active TB are assumed to undergo diagnostic attempts at a defined rate. Unlike other models that assume diagnostic attempts to reflect tests with a single diagnostic or defined series of diagnostic tests, our conceptualization of “diagnostic attempt” is more inclusive and incorporates all initial and follow-on testing that is performed until a diagnosis of TB is either made or excluded by the diagnosing practitioner or team of practitioners . One diagnostic attempt may include clinical judgment, radiography and other tests in addition to the diagnostic test for TB specifically (smear microscopy or molecular test) and is considered to lead to a diagnosis (Table S1). By using this more inclusive definition of “diagnostic attempt,” we maximize our ability to account for empiric treatment but may underestimate the impact of a rapid diagnostic test in terms of reducing diagnostic delays, which are intrinsically incorporated into our rate of diagnostic attempt. We assumed that 85% of patients that were diagnosed were going to receive treatment.

At the time of diagnosis and treatment, patients with active TB are instantaneously placed into one of three subpopulations:

1. Cured/Recovered: Those who are cured from TB whether or not that completed a full course of therapy.
2. Active, previously treated TB: Those who default or complete therapy but will relapse.
3. Failure: Those who fail therapy.

Depending on the baseline susceptibility of the strain (e.g. INH-resistant) the patients may also develop further resistance. Patients who develop additional resistance mutations are assigned directly to the failing group in the respective drug-resistant compartment (e.g. MDR resistance). The distribution into the subpopulation compartments (cured, previously treated active TB, failure and resistance) reflects the percentages as reported in the literature (Table S2).

We presumed that, before year zero, drug-susceptibility testing (DST) is limited only to those who have failed a previous course of TB therapy and remain symptomatic . In all other cases, patients are treated with standard short-course (first-line) therapy. If resistance is present at initial diagnosis, a higher proportion of patients with fail, recur or develop further resistance (Table S2). Patients who already failed therapy are assumed to receive second-line therapy for MDR-TB after the duration that it takes for them to be identified as failing first-line therapy (six to eight months).

Patients with recurrent TB after completing an initial course of therapy are assumed to be diagnosed at the same rate as new cases. The likelihood of being diagnosed depends on the diagnostic method available . If the patient does not receive DST or the DST does not diagnose resistance, treatment including an aminoglycoside and lasting a total of eight months (“category II”) is assumed. In contrast, if the patient is diagnosed with a resistance mutation based on DST, a second-line regimen is assumed, with correspondingly higher cure rates (Table S2). Patients who fail therapy are assumed to be re-diagnosed at a rate corresponding to the duration of their initial treatment course.

All patients with active TB are considered infectious. Patients who are failing but on partially active therapy (i.e. 1 or 2 active drugs) are assumed to be less infectious than untreated patients . Smear-negative cases are responsible for about 20% of cases in contact and outbreak investigations . Active regimens immediately render the patient noninfectious and return the patient’s mortality risk to that of an uninfected individual . Patients who are cured return to a susceptible state but are considered to have partial protection against reinfection similar to that of latent TB infection . If these patients acquire infection again, they progress to the previously-treated active TB group.

**Table S1: Parameter estimates**

| **Parameter** | **Value** | **Range** | **Reference** |
| --- | --- | --- | --- |
| Birth/non-TB death rate per year (life expectancy 60 years) | 0.017 | 0.015-0.018 |  |
| TB mortality per year | 0.15 | 0.10-0.22 |  |
| Attenuation of infectiousness with INH resistance mutation | 0. 986 | 0.85-1.0 |  |
| Attenuation of infectiousness with MDR resistance mutation | 0.772 | 0.6-0.923 |  |
| Relative infectivity of smear-negative TB infectious person-year or partially treated person | 0.2 | 0.16–0.28 |  |
| Relative protection from reinfection in latent/recovered TB | 0.45 | 0.4-0.55 |  |
| Proportion of TB infections progressing rapidly to active TB | 0.14 | 0.05-0.14 |  |
| Endogenous reactivation rate per year | 0.0005 | 0.08-1.4 x10^-3^ |  |
| Rate of self-cure in active TB per year | 0.1 | 0.08-0.25 |  |
| Percent of patients without access to diagnostics | 15 | 5-25 |  |
| Percent of resistant cases in 2010, new cases   - INH monoresistant - Multi-drug resistant | 15.0  2.1 |  |  |
| Sensitivity of current diagnostic standard per diagnostic attempt | 0.80 | 0.6-0.9 |  |
| Sensitivity of molecular methods per diagnostic attempt | 0.95 | 0.75-0.98 |  |
| Sensitivity of molecular methods for RIF resistance (as a marker of MDR) | 0.94 | 0.90-0.96 |  |
| Sensitivity of molecular methods for INH resistance detecting katG (high-level resistance) and inhA (low-level resistance) | 0.88 | 0.7-0.95 |  |
| Sensitivity of culture-based methods for RIF and INH resistance | 1 |  | Assumed |
| Duration of illness before diagnostic attempt completed with molecular test (months) | 6 | 4-12 |  |
| Duration of failing therapy before diagnostic attempt completed with molecular test (months) | 3 | 2-6 |  |
| Proportional change in diagnostic rate using standard diagnosis compared to molecular diagnosis | 0.75 |  |  |

**Table S2: Estimates of treatment success rates**

| Patients with new infection on standard short-course therapy | | | |
| --- | --- | --- | --- |
| Patients with sensitive TB. Proportions:  Cured  Recurrence (default + relapse)  Failing  Developing INH resistance  Developing MDR resistance | 0.88  0.09  0.025  0.004  0.001 | 0.75-0.95  0.02-0.1  0.01-0.03  0.003-0.01  0.0005-0.005 |  |
| Patients with MDR TB treated with standard short-course therapy (DST not done). Proportions:  Cured  Recurrence (default + relapse)  Failing (not due to new drug resistance) | 0.25  0.35  0.40 | 0.2-0.4  0.10-0.50  0.3-0.70 |  |
| Patients with INH- monoresistant TB treated with standard short-course therapy (DST not done). Proportions:  Cured  Recurrence (default + relapse)  Failing (not due to new drug resistance)  Developing MDR resistance | 0.80  0.09  0.10  0.01 | 0.65-0.90  0.05-0.2  0.03-0.2  0.001-0.02 |  |
| Patients with new infection on therapy based on DST | | | |
| Patients with MDR TB on active therapy based on DST. Proportions:  Cured  Recurrence (default + relapse)  Failing | 0.60  0.22  0.18 | 0.40-0.83  0.1-0.30  0.1-0.30 |  |
| Patients with INH-monoresistant TB on active therapy based on DST. Proportions:  Cured  Recurrence (default + relapse)  Failing (not due to new drug resistance)  Developing MDR resistance | 0.88  0.09  0.029  0.001 | 0.75-0.95  0.05-0.17  0.02-0.11  0.001-0.005 |  |

**Table S3: Model compartments**

| **Compartment** | **Description** |
| --- | --- |
| *S* | Susceptible never infected before -  Maximum risk of TB infection |
| *L_d_* | Latently infected -  Offers partial protection against re-infection |
| *A_d_* | New, actively infected that will be diagnosed and treated -  Infectious, increased mortality |
| *N_d_* | New, actively infected but without access to diagnostics -  Infectious, increased mortality |
| *F_d_* | Failure – requiring ongoing therapy -  Individuals who develop resistance directly go from active treatment into the respective failed resistant compartment  Infectious at the rate of smear –negative cases |
| *R_d_* | Active, previously treated -  Individuals who require retreatment because they default, relapse or acquire reinfection |
| *C_d_* | Cured/Recovered -  At risk for recurrent infection with partial infection conferred by prior infection |

Legend: *d* refers to drug susceptibility (sensitive (s), multidrug-resistant (MDR), or INH-monoresistant

1. **Description of parameters**

This section provides a more detailed description of the primary parameters for which the most data exist to inform parameter estimates. The estimates for parameters with ranges and citations are listed in Table S1. The values chosen for the sensitivity analysis are reported in Table S4.

The population size of the hypothetical model population is set at 100,000. Individuals enter the model at birth, being HIV-negative and uninfected with TB. They exit the model upon dying or reaching their 60th birthday. Mortality rates depend on an individual’s TB status. In patients with active TB a mortality rate of 0.15/year (average for both smear negative and smear positive; incorporating an early, subclinical phase) is added to the mortality of the uninfected. Patients who are partially treated (i.e. only 1 or 2 active drugs) are considered to have the same mortality rate than patients who have smear-negative TB (25% of smear-positive TB).

For simplicity, we assumed no HIV infection (as adult HIV prevalence in India is 0.4%) and no INH preventive therapy (IPT, currently under the Revised National TB Control Program [RNTCP] only recommended for close contacts with HIV infection and children less than 5 years of age) .

The transmission rate (β) denotes the number of secondary infections per infectious case. We calculate the transmission based on the TB incidence in India in 2011 (181/100,000) . We perform a sensitivity analysis to account for a possible under- or overestimated TB incidence. Assuming an increase in resistance since introduction of anti-mycobacterial therapy in the 1950s, an attenuation of infectivity has to be assumed for MDR strains to explain the currently observed MDR estimates. Similar results have also been shown in laboratory experiments (with an attenuation for MDR strains estimated around 0.73) . Laboratory experiments on the transmissibility of INH-monoresistant TB suggest less attenuation and some publications even find an enhanced transmissibility (range from 0.7 to 1.1) . In our model, we estimate the attenuation of MDR and INH-monoresistant strains by reproducing the trajectory of MDR and INH since 1950 to reach the proportions known for India in 2010. However, given the possibility of compensatory mutations that restore the transmissibility, we do a sensitivity analysis around these parameters. We also specifically model two scenarios where a compensatory mutation would result either in doubling of MDR cases or in a 25% increase in INH-monoresistant cases in year 10 (see additional analyses below).

The proportion of TB infections that progresses rapidly to active TB is taken as the proportion of patients who develop active TB within one year of TB infection from Vynnycky and Fine’s estimation in a British Population . Of note, this estimate of 14% is greater than the classically assumed 5%, or half of a 10% lifetime risk for active TB if infected in childhood. Vynnycky and Fine suggest that the risk of rapid progression is higher in adults (14%) than in children (4%). To account for the possibility of overestimating this parameter, we perform a univariate sensitivity analysis to a lower bound of 5%.

The percentage of patients who are never diagnosed due lack of access to care also is a matter of debate. Data exists from hospital studies primarily in an HIV-positive population where up to three fourth of patients die of TB and a quarter was never suspected to have TB prior to dying . The proportion might be even higher in patients dying in the community but studies are limited . However, these estimates do not take into account self-cure and estimates are certainly presumed to be lower in HIV-negative patients . A sensitivity analysis was done to a lower limit of 5% and an upper limit of 25% to account for uncertainty in this parameter value.

The annual endogenous reactivation rate in HIV-negative patients is taken from Ferebee’s 1970 review of TB chemoprophylaxis trials . The estimate of the percent of patients that self cure is taken from prior work of Enarson and Rouillon .

The diagnostic rate is calculated as the inverse of the mean time to initial diagnosis, which is the sum of the disease duration of untreated TB and the provider delay after presentation. The mean time to diagnosis varies between studies . Given that the estimate may affect the calculated TB incidence significantly, we perform a sensitivity analysis to account for a range of duration until diagnosis (Table S4). We assumed that 85% of patients that were diagnosed were going to receive treatment.

The sensitivity of TB detection with established methods can be estimated from case detection rates in the recent WHO report . For the Xpert MTB/RIF, accuracy estimates have been published in the demonstration studies by Boehme et al. and a recent meta-analysis by Steingart et al. . The accuracy of molecular testing for rifampin has also been well described in these studies . For INH molecular resistance testing the sensitivity depends on which mutations are targeted. The most common mutation, katG, is responsible for high-level INH resistance . Tests that capture the katG mutation only, will detect only about 84% of INH resistance. Additional detection of inhA mutation results in a sensitivity of about 88%, however the importance of the low-level resistance conferred by inhA on clinical outcomes is debated . For our model, we use 88% sensitivity for both katG and inhA detection as the primary estimate. Additional mutations conferring reduced INH sensitivity are known but are more difficult to incorporate in tests and the effect of these mutations on clinical outcomes has not been well described . Advances in test development might enhance the accuracy of detection and resistance testing with molecular testing in the near future; therefore a sensitivity analysis is done assuming the tests might reach perfect sensitivity.

Treatment success estimates are taken from the most recent WHO Global TB control report and other publications as outlined in the table. The proportion of patients with INH mono-resistance who are cured when treated with standard short-course therapy is highly debated in the literature . The discrepancies in the literature are probably due to varying diagnostic methods to confirm resistance (with varying reliability) as well as different mutations conferring different levels of resistance, which vary in their clinical importance. We chose a primary estimate to reflect a more pessimistic estimation with high failure and resistance development (80% cure rate and 1% probability of acquiring MDR-TB under standard therapy). As this is a key outcome parameter in our model, a sensitivity analysis is done that incorporates a wide range of estimations . The proportion of treatment success of INH-resistant TB with DST-guided therapy was set at the upper limit of a conceivable range in the base-case scenario (which results in INH-resistant TB being treated with the same likelihood of success than drug-susceptible TB), to allow for a test that detects INH resistance to have the highest possible effect.

We conducted sensitivity analyses as outlined in the methods across ranges as outlined in Table S4. Furthermore, to estimate variability associated with simultaneous changes in all parameters, we also conducted a probabilistic uncertainty analysis, using Latin Hypercube Sampling to select values randomly from beta distributions (for parameters, e.g. probabilities, bounded from 0 to 1) or gamma distributions (for parameters, e.g. rates, bounded from 0 to infinity) for each parameter across a range of 25% unless otherwise indicated. Simulations that caused a two-fold increase or 50% decrease in TB incidence over 10 years were rejected. We conducted more than 10,000 independent simulations in this fashion, thus generating 95% uncertainty ranges, defined as the intervals bounded by the 2.5 and 97.5 percentiles of all acceptable simulations.

**Table S4: Univariate sensitivity analysis – base-case value and range**

| **Parameter** | **Value** | **Range** |
| --- | --- | --- |
| Birth/non-TB death rate per year (life expectancy 60 years) | 0.017 | 0.015-0.018 |
| TB mortality per year | 0.15 | 0.10-0.22 |
| Attenuation of infectiousness by INH resistance mutation before study starts | 0. 986 | 0.85-1.0 |
| Attenuation of infectiousness by MDR resistance mutation before study starts | 0.772 | 0.6-0.923 |
| Relative protection from reinfection in latent/recovered TB | 0.45 | 0.4-0.55 |
| Proportion of TB infections progressing rapidly to active TB | 0.14 | 0.05-0.14 |
| Endogenous reactivation rate per year | 0.0005 | 0.08-1.4 x10^-3^ |
| Rate of self-cure in active TB per year | 0.1 | 0.08-0.25 |
| Percent of patients without access to diagnostics | 15 | 5-25 |
| Sensitivity of current diagnostic standard per diagnostic attempt | 0.80 | 0.6-0.9 |
| Sensitivity of molecular methods per diagnostic attempt | 0.95 | 0.75-1.0 |
| Sensitivity of molecular methods for INH resistance detecting katG (high-level resistance) and inhA (low-level resistance) | 0.88 | 0.7-1.0 |
| Duration of illness before diagnostic attempt completed with molecular test (months) | 6 | 4-12 |
| Duration of failing therapy before diagnostic attempt completed with molecular test (months) | 3 | 2-6 |
| INH-monoresistant TB cured with standard short-course therapy | 0.80 | 0.65-0.88* |
| Probability of development of MDR resistance among patients failing standard short-course therapy | 0.09 | 0.009-0.18 |
| INH-monoresistant TB cured with DST-guided therapy | 0.88 | 0.75-0.88* |
| Probability of development of MDR resistance among patients failing DST-guided therapy | 0.033 | 0.033-0.33 |
| Attenuation in treatment success with retreatment in patients who failed, defaulted, relapsed or acquired reinfection | 0.9 | 0.80-1.0 |

*upper bound defined by cure rate of drug-sensitive TB

1. **Model parameters and there symbolic representation**

| **Parameter** | **** | **Baseline Value** |
| --- | --- | --- |
| Transmission rate (transmission events per infectious person-year) | *β* | 6.50 (solved) |
| Transmission rate (transmission events per infectious person-year) for multi-drug resistant TB | *β__* | Derived (below) |
| Transmission rate (transmission events per infectious person-year) for INH resistant TB | *β__* | Derived (below) |
| Attenuation of infectiousness by MDR resistance mutation | *c_MDR_* | 0.772 (solved) |
| Attenuation of infectiousness by INH resistance mutation | *c__* | 0.986 (solved) |
| Force of infection | *λ* | Derived (below) |
| Endogenous reactivation rate, per year | *ε* | Table S1 |
| Proportion of infections progressing rapidly to active TB | *π* | Table S1 |
| Relative protection from reinfection in latent/recovered TB | *ι* | Table S1 |
| TB mortality rate, per year | *μ__* | Table S1 |
| Spontaneous cure rate, per year | *υ* | Table S1 |
| Baseline mortality rate, per year | *μ__* | Table S1 |
| Relative transmission rate (per year), failing cases | *σ* | Table S1 |
| Duration of illness before diagnostic attempt completed | *δ_nr_* | 6 |
| Duration of failing therapy before diagnostic attempt completed with molecular test (months) | *δ_fail_* | 3 |
| Diagnostic rate for new or default/relapse or reinfection cases | *τ_r_* | Derived (below) |
| Diagnostic rate for failure cases | *τ__* | Derived (below) |
| Proportion of patients without access to diagnostics | *η* | Table S1 |
| Probability of receiving a molecular diagnostic test as a new case | *ω__* | Dependent on scenario |
| Probability of receiving a molecular diagnostic test as a retreatment case | *ω_re_* | Dependent on scenario |
| Probability of receiving a molecular diagnostic test when failing therapy | *ω_fail_* | Dependent on scenario |
| Proportion of patients initiating treatment after diagnosis | *κ* | Table S2 |
| Probability of cure | *δ_χ_* | Table S2 |
| Probability of default/relapse | *δ_def_* | Table S2 |
| Probability of failure without resistance development | *δ_fail_* | Table S2 |
| Probability of failure with development of INH resistance | *δ_INH_* | Table S2 |
| Probability of failure with development of multi-drug resistance | *δ_MDR_* | Table S2 |
| Probability of cure in default/relapse or reinfection cases | *δ_re/c_* | Table S2 |
| Probability of default/relapse in default/relapse or reinfection cases | *δ_re/def_* | Table S2 |
| Probability of failure without resistance development in default/relapse or reinfection cases | *δ_re/fail_* | Table S2 |
| Probability of failure with development of INH resistance in default/relapse or reinfection cases | *δ_re/INH_* | Table S2 |
| Probability of failure with development of multi-drug resistance in default/relapse or reinfection cases | *δ_re/MDR_* | Table S2 |
| Probability of cure in individuals failing therapy | *δ_fail/c_* | Table S2 |
| Probability of default/relapse in individuals failing therapy | *δ_fail/def_* | Table S2 |
| Probability of failure without resistance development in individuals failing therapy | *δ_fail/fail_* | Table S2 |
| Probability of failure with development of INH resistance in individuals failing therapy | *δ_fail/INH_* | Table S2 |
| Probability of failure with development of multi-drug resistance in individuals failing therapy | *δ_fail/MDR_* | Table S2 |

**Derived parameters**

1. Transmission rate for resistant strains:

The transmission rate for resistant strains is a function of the attenuation of the individual strains and the transmission rate (β. The attenuation (c_d_; where d indicates the drug susceptibility) varies for different strains. While the current epidemic of MDR can only be explained by a significant attenuation of the strain, the level of attenuation of INH-resistant strains is less clear (further discussion in the methods section). The level of attenuation was solved for the observed proportion of resistance in the population in year 2011 in India.

MDR: *β__* = *β* *  *c_MDR_*

INH: *β_INH_* = *β* *  *c_INH_*

1. Diagnostic and treatment rate:

The diagnostic rate is defined as the inverse of the mean time to initial diagnosis. The time to initial diagnosis depends on the case category of the patient (failing versus new/relapse) and the diagnostic test the patient receives. Failing cases are in the system already also probably have more pronounced symptoms and are therefore more likely to be diagnosed faster. The time to diagnosis for new and relapse cases incorporates a subclinical period where the patient is infectious but not seeking care yet. Once diagnosed only a proportion of patients (κ) actually initiates treatment while others are lost to follow up.

 active * τ_r_* = 1/*δ_nr_* * (*ω__**1 + (1-*ω__*)*0.75)* κ

Active, previously treated cases: *τ_r_* = 1/*δ_nr_* * (*ω_re_* *1 + (1-*ω_re_*)*0.75) * κ

Failure  *τ__* = 1/*δ_fail_* * (*ω_fail_* *1 + (1-*ω_fail_*)*0.75) * κ

1. Force of Infection (λ)

TB infection is modeled as a density-dependent process, a function of the transmission rate (β), attenuated if the source case is a resistant case (see transmission rates above; subscript d indicates drug susceptibility), number of individuals with infectious TB (*A_d_*, active new cases; *R_d_*, active, previously treated cases; *F_d_*, individuals failing therapy), and divided by the total size of the population. Failure cases are presumed to have an attenuated infectivity on the level of a smear-negative case due to partial treatment (σ) .

λ_d_ = β * c*_INH/MDR_* * (A_d_ + R_d_+ N_d_+ σ * F_d_) / (S_d_ + L_d_ + A_d_ + F_d_ + C_d_ + R_d_)

1. **Model Equations**

Rates of flow between compartments are governed by the system of ordinary differential equations listed in equations 2-6 for drug-sensitive TB, with equation one being the susceptible compartment that is not subdivided by drug-sensitivity. The model is programmed in Python, and the source code for the model is available on the website Github (https://github.com/cdenki/INH_RIF_model_PLOSone.git).

Equation 1. Susceptible Compartment (S)

dS[t]/dt = [μ_bl_* (S + L_d_ + A_d_ + F_d_ + C_d_ + R_d_) + μ_TB_ * (A_d_ + R_d_+ N_d_)] ‒ (λ_d_ + μ_bl_)* S

where [μ_bl_ * (S + L_d_ + A_d_ + F_d_ + C_d_ + R_d_) + μ_TB_ * (A_d_ + R_d_+ N_d_)] is the sum of all mortality (to maintain a constant population), λ_d_ is the force of infection for all different types of TB (drug-susceptible, MDR, INH-resistant), μ_TB_ is the TB related mortality in the active cases and μ_bl_ is the non-TB mortality rate. Thus, uninfected individuals leave this compartment through infection and death, and the compartment is replenished at a rate that matches total mortality and thereby maintains a constant population. This compartment is not subdivided by drug-sensitivity.

Equation 2. Latently Infected Compartment (L)

dL_s_[t]/dt = [λ_s_ * (1 ‒ π) * S + λ_s_ * (1 ‒ π) * (1 - ι)* L_d_ + υ  (_S_ + _S_)]* *‒ [λ_d_ * (1 ‒ ι) + ε + μ_bl_] * L_s_

where λ_s_ is the force of infection for drug-susceptible TB and λ_d_ is the force of infection for all different types of TB (drug-susceptible, MDR, INH-resistant), π is the proportion of recent infections that progress rapidly to active TB from susceptible and different latent department (independent of primary dominant strain), ι is the relative reduction in rapid progression after infection among people with latent TB, υ is the rate of self-cure, ε is the endogenous reactivation rate, and μ_bl_ is the non-TB mortality rate. Thus, susceptible individuals who get infected (newly or with a different strain) but do not progress rapidly to active disease are modeled as having latent infection, and latently- infected individuals leave this compartment through TB reinfection (with rapid progression they go into respective active compartments; without rapid progression they go into respective latent compartments), endogenous reactivation, and death.

Equation 3. Active TB Compartment (A)

dA_s_[t]/dt = [λ_s_ * π * (1 – η) * S + λ_s_ * π * (1 - ι) * (1 – η) * L_d_ + ε  L_s_]* *‒ [*τ_r_* * (δ_χ_ + δ_def_ + δ_fail_ + δ_INH_ + δ_MDR_) + υ + μ_bl_ + μ_TB_] * A_s_

where λ_s_ is the force of infection for drug-susceptible TB, π is the proportion of recent infections that progress rapidly to active TB, ι is the relative reduction in rapid progression after infection among people with latent TB, η is the proportion without access to diagnostics, ε is the endogenous reactivation rate, *τ_r_* is the diagnostic rate for new infections, δ is the probability of cure (δ_χ_), default/relapse(δ_def_), failure (δ_fail_) or resistance development (δ_INH_, δ_MDR_) with diagnosis and treatment in new cases, υ is the rate of self-cure, μ_bl_ is the non-TB mortality rate, and μ_TB_ is the TB mortality rate. Thus, susceptible individuals and latently infected individuals (those not protected through prior infection) who progress rapidly into active infection as well as those who reactivate constitute the active infection compartment. Individuals leave the compartment through diagnosis at a defined diagnostic rate and treatment resulting in cure, default/relapse, failure or development of resistance, spontaneous cure, or death (from TB or other causes).

Equation 4. Never-diagnosed, active compartment (N)

dN_s_[t]/dt = [λ_s_ * π * η * S + λ_s_ * π * (1 - ι) * η * L_d_ + ε  L_s_]* *‒ [υ + μ_bl_ + μ_TB_] * N_s_

where λ_s_ is the force of infection for drug-susceptible TB, π is the proportion of recent infections that progress rapidly to active TB, ι is the relative reduction in rapid progression after infection among people with latent TB, η is the proportion without access to diagnostics, ε is the endogenous reactivation rate, υ is the rate of self-cure, μ_bl_ is the non-TB mortality rate, and μ_TB_ is the TB mortality rate. Thus, susceptible individuals and latently infected individuals (those not protected through prior infection) who progress rapidly into active infection as well as those who reactivate and never get diagnosed due to lack of access to diagnostics constitute the active never-diagnosed compartment. Individuals leave the compartment only through spontaneous cure, or death (from TB or other causes).

Equation 5: Active, previously treated cases (R)

dR_s_[t]/dt = [*τ_r_* * δ_def_ * A_s_ + *τ_fail_* * δ_fail/def_ * F_s_ + *τ_r_* * δ_re/def_ * R_s_ + λ_s_ * π * (1 - ι) * _d_ ] – [*τ_r_* * (δ_re/χ_ + δ _re/def_ + δ _re/fail_ + δ _re/INH_ + δ_re/MDR_) + υ + μ_bl_ + μ_TB_] * R_s_

where *τ_r_* is the diagnostic and treatment rate for new infections, δ_def_ is the probability of default/relapse in new cases, *τ_fail_* is the diagnostic and treatment rate for individuals failing therapy, δ_fail/def_ is the probability of default/relapse in failing cases, δ_re/def_ is the probability of default/relapse in active, previously treated cases, λ_s_ is the force of infection for drug-susceptible TB, π is the proportion of recent infections that progress rapidly to active TB out of different cured compartments independent of drug-susceptibility, ι is the relative reduction in rapid progression after infection among people with latent TB, δ_re_ is the probability of cure, default/relapse, failure or resistance development with diagnosis and treatment in active, previously treated cases, υ is the rate of self-cure, μ_bl_ is the non-TB mortality rate, and μ_TB_ is the TB mortality rate. Thus, individuals enter the compartment through relapse or default out of the active new (A_d_), active previously treated (R_d_) or failure (F_d_) compartments or through reinfection of patients who had achieved cure from a prior infection (C_d_). Individuals leave the compartment through diagnosis at a defined diagnostic rate and treatment resulting in cure, default/relapse, failure or development of resistance, spontaneous cure, or death (from TB or other causes).

Equation 6: Failure (F)

dF_s_[t]/dt = [*τ_r_* * δ_fail_ * A_s_ + *τ_r_* * δ_re/fail_ * R_s_ + *τ_fail_* * δ_fail/fail_ * F_s_] – [*τ_fail_* * (δ_fail/χ_ + δ _fail/def_ + δ _fail/fail_ + δ _fail/INH_ + δ_fail/MDR_) + μ_bl_] * F_s_

where *τ_r_* is the diagnostic and treatment rate for new infections, δ_fail_ and δ_re/fail_ are the probability of failure in active new and previously treated cases, *τ_fail_* is the diagnostic and treatment rate for individuals failing therapy, δ_fail/fail_ is the probability of failure with a change in therapy (to category II treatment or treatment guided by drug-susceptibility) in failing cases, δ_fail_ is the probability of cure, default/relapse, failure or resistance development with diagnosis and treatment in failure cases, and μ_bl_ is the non-TB mortality rate. Thus, individuals enter the compartment through failing therapy for a new or failing infection (A_d_ and F_d_ respectively) or failing retreatment (R_d_). Individuals leave the compartment through diagnosis at a defined diagnostic rate and treatment resulting in cure, default/relapse, failure or development of resistance, or death (from other causes).

Equation 7: Recovered/Cured Compartment (C)

dR_s_[t]/dt = [*τ_r_* * δ_c_* A_s_ + *τ_r_* * δ_re/c_ * R_s_ + *τ_fail_* * δ_fail/c_ * F_s_ + λ_s_ * (1 - π) * (1 - ι) * _d_ + υ] – [λ_d_ * (1 - ι) + μ_bl_] * C_s_

where *τ_r_* is the diagnostic and treatment rate for new infections, δ_c_ is the probability of cure in new cases, *τ_fail_* is the diagnostic and treatment rate for individuals failing therapy, δ_fail/c_ is the probability of cure in failing cases, δ_re/c_ is the probability of cure in active, previously treated cases, λ_s_ is the force of infection for susceptible TB and λ_d_ is the force of infection for all TB, π is the proportion of recent infections that progress rapidly to active TB, ι is the relative reduction in rapid progression after infection among people with latent TB, υ is the rate of self-cure and μ_bl_ is the non-TB mortality rate. Thus, individuals enter the compartment through being cured out of the active new (A_d_), active, previously treated (R_d_) or failure (F_d_) compartments or through reinfection of patients who had achieved cure from a prior infection (C_d_) but do not progress to active disease. Individuals leave the compartment through TB reinfection or death (from TB or other causes).

Equations for drug-resistant TB are similar except for lack for further resistance development in MDR and lack of INH-resistance development in already INH-resistant strains.

1. **Additional analyses**

We performed additional analyses to assess whether our finding hold up if certain assumptions about the prevalence of INH resistant or MDR-TB were not true or if the epidemic would change dramatically over the ten years (i.e. through increased transmissibility of the drug-resistant TB because of compensatory mutations).

First, we assumed that a compensatory mutation were to occur in INH-resistant strains at year zero that renders the strains equally transmissible as drug-sensitive TB. In this scenario, the proportion of INH resistance cases among total cases would increase from 15.2% at year zero to 16.2% after 10 years. For a 25% increase in INH resistance cases to occur, a compensatory mutation and a reduction in the proportion cured to only 40% with standard therapy would have to be assumed. In this very unrealistic but illustrative scenario, a test for INH resistance detection in addition to rifampin resistance detection would result in a 19% reduction of the proportion of INH monoresistant cases from 25.0% to 20.0%; Figure S1D) and 7 less cases per 100,000 in year ten of a high-coverage implementation (Figure S1B). However even under this “worst-case scenario”, MDR would only be reduced by an additional 10% compared to a scenario with TB + RIF testing only, with the number of cases being reduced by 0.9 per 100,000 (Figure S1A and S1C).

Similarly, even under extreme assumptions such as under-reporting INH-monoresistant TB to the WHO by a factor of two at year zero or the doubling of MDR through a compensatory mutation over the next ten years the proportional impact of including susceptibility testing for INH-resistance was projected to be similar. Figure S2 and S3 show corresponding trends in MDR-TB and INH-monoresistant TB cases in the high coverage setting as the absolute number of cases detected per 100,000 (A, B) and as a proportion of all cases (new cases and relapse/default cases) detected (C, D) in these scenarios.

Projections over 50 years (albeit unrealistic as conditions after 10 years are likely going to change) confirm the trend projected in the first 10 years, although the effect of the diagnostic tests levels off (Figure S4 and S5).

A separate sensitivity analysis explored the boundaries of possible implications of mixed infections by considering the alternative scenarios that, upon reinfection, the superinfecting strain always and never becomes the dominant strain. While we observed an added benefit of an RIF+INH resistance test in addition to detection of Rifampin resistance alone in the scenario where no protection from reinfection was present, the overall benefit both on the total number of MDR and INH-resistant TB detected was small (Figure S6). Lastly, we explored a 3^rd^ scenario in which mixed infections always behave according to the highest level of drug resistance (data not shown). None of these assumptions change the primary conclusions regarding the incremental transmission value of testing for INH.

**Supporting Information Legends**

**Figure S1: Impact of resistance testing with a hypothetical increase of INH**

Assuming a 25% increase of INH cases among all cases detected in 2020 (through a compensatory mutation with equal transmissibility compared to sensitive cases and by presuming a cure rate with standard therapy of only 40%), the impact of a TB+RIF on the number of MDR cases among total cases detected and the proportion of MDR would be similar (A and C) compared to data shown in Figure 3, however the effect of a test for INH resistance would be somewhat enhanced. The proportion INH monoresistant at year 10 would be reduced from 23.7% to 18.5% in the TB+RIF to the TB+RIF/INH scenario (D), with a reduction of INH resistant cases by 8/100,000 (B). The proportion MDR would be 0.7% lower (C), with a total reduction of 1 MDR case per 100,000 (A). Note that in panel B the lines for scenarios with a molecular test only and a test that detects rifampin resistance are overlapping.

**Figure S2: Impact of resistance testing with a hypothetical increase of MDR**

Assuming a doubling of multi-drug resistant (MDR) cases among all cases detected by year 10 (through a compensatory mutation that substantially increases transmissibility of MDR cases), the impact of a test for rifampin resistance would be more substantial (A and C) compared to a test for TB detection only; however, the additional effect of an INH resistance test on INH and MDR resistance would remain small. The proportion INH resistant cases at year 10 would be further reduced by 4.7% compared to the TB+RIF scenario (D), with a reduction of INH resistant cases by 6.5/100,000 in year ten (B). The proportion MDR would be decreased by 0.6% over a TB+RIF scenario (C), with a reduction in MDR cases of 1.6 per 100,000 (D). Note that in panel B the lines for the two scenarios TB detection only and TB+RIF are overlapping.

**Figure S3: Impact of resistance testing with a higher INH prevalence**

Assuming that isoniazid (INH) resistance is underreported and it is in fact double as common as reported in year zero (30% instead 15% in new cases), the addition of a test for INH resistance still would have only a small effect on reducing the proportion of INH resistant cases and multi-drug resistant cases beyond that of a molecular test with rifampin resistance testing alone (A and B). The proportional impact of a TB+RIF scenario (C and D) would be similar to the scenario reported in 3C and 3D.

**Figure S4: Impact of resistance testing on incidence and mortality over 50 years**

Trajectory of overall TB incidence (solid lines, left axis) and mortality (dotted lines, right axis) over 50 years with introduction of a molecular test for diagnosis and detection of rifampin (RIF) resistance, with or without a molecular test for isoniazid (INH) resistance. Grey lines correspond to the high-coverage scenario (i.e. 50%, 80% and 100% coverage among new, previously treated and failure cases, respectively, excluding those with no access to care), green lines to an alternative lower-coverage scenario (15%, 25%, and 30% among new, previously treated and failure). The curves for TB+RIF versus TB+RIF/INH are indistinguishable on the graph because the projected outcomes of incidence and mortality are so similar.

**Figure S5: Impact of resistance testing on multi-drug and isoniazid resistance over 50 years**

Projected trajectories for multi-drug resistant (MDR) (A, C) and INH-resistance (INHr) (B, D) cases with TB detection, TB+RIF and TB+RIF/INH over 50 years. Results are shown as the absolute number of MDR or INHr cases per 100,000 (A, B) and as a proportion of all cases (new cases and relapse/default cases) detected (C, D).

**Figure S6: Impact of resistance testing on multi-drug and isoniazid resistance over 10 years under different assumptions relating to superinfection**

Projected trajectories for multi-drug resistant (MDR) (A) and INH-resistance (INHr) (B) cases with TB detection, TB+RIF and TB+RIF/INH over 10 years. Results are shown as the absolute number of MDR or INHr cases per 100,000 for the high-coverage scenario with complete protection against superinfection versus no protection at all (i.e. superinfecting strain becomes the dominant strain).

**References**

1. Andrews JR, Noubary F, Walensky RP, Cerda R, Losina E, et al. (2012) Risk of progression to active tuberculosis following reinfection with Mycobacterium tuberculosis. Clin Infect Dis 54: 784-791.

2. Vynnycky E, Fine PE (1997) The natural history of tuberculosis: the implications of age-dependent risks of disease and the role of reinfection. Epidemiol Infect 119: 183-201.

3. Sutherland I, Svandova E, Radhakrishna S (1982) The development of clinical tuberculosis following infection with tubercle bacilli. 1. A theoretical model for the development of clinical tuberculosis following infection, linking from data on the risk of tuberculous infection and the incidence of clinical tuberculosis in the Netherlands. Tubercle 63: 255-268.

4. van Leth F, van der Werf MJ, Borgdorff MW (2008) Prevalence of tuberculous infection and incidence of tuberculosis: a re-assessment of the Styblo rule. Bull World Health Organ 86: 20-26.

5. Keeler E, Perkins MD, Small P, Hanson C, Reed S, et al. (2006) Reducing the global burden of tuberculosis: the contribution of improved diagnostics. Nature 444 Suppl 1: 49-57.

6. Vassall A, van Kampen S, Sohn H, Michael JS, John KR, et al. (2011) Rapid diagnosis of tuberculosis with the Xpert MTB/RIF assay in high burden countries: a cost-effectiveness analysis. PLoS Med 8: e1001120.

7. World Health Organization (2012) Global tuberculosis report 2012. Geneva pp. 1-282.

8. Behr MA, Warren SA, Salamon H, Hopewell PC, Ponce de Leon A, et al. (1999) Transmission of Mycobacterium tuberculosis from patients smear-negative for acid-fast bacilli. Lancet 353: 444-449.

9. Fennelly KP, Jones-Lopez EC, Ayakaka I, Kim S, Menyha H, et al. (2012) Variability of infectious aerosols produced during coughing by patients with pulmonary tuberculosis. Am J Respir Crit Care Med 186: 450-457.

10. World Health Organization (2011) Global tuberculosis report 2011. Geneva pp. 1-246.

11. Pym AS, Saint-Joanis B, Cole ST (2002) Effect of katG mutations on the virulence of Mycobacterium tuberculosis and the implication for transmission in humans. Infect Immun 70: 4955-4960.

12. van Soolingen D, de Haas PE, van Doorn HR, Kuijper E, Rinder H, et al. (2000) Mutations at amino acid position 315 of the katG gene are associated with high-level resistance to isoniazid, other drug resistance, and successful transmission of Mycobacterium tuberculosis in the Netherlands. J Infect Dis 182: 1788-1790.

13. Cohen T, Murray M (2004) Modeling epidemics of multidrug-resistant M. tuberculosis of heterogeneous fitness. Nat Med 10: 1117-1121.

14. Billington OJ, McHugh TD, Gillespie SH (1999) Physiological cost of rifampin resistance induced in vitro in Mycobacterium tuberculosis. Antimicrob Agents Chemother 43: 1866-1869.

15. Gagneux S, Long CD, Small PM, Van T, Schoolnik GK, et al. (2006) The competitive cost of antibiotic resistance in Mycobacterium tuberculosis. Science 312: 1944-1946.

16. Ferebee SH (1970) Controlled chemoprophylaxis trials in tuberculosis. A general review. Bibl Tuberc 26: 28-106.

17. Grzybowski S, Enarson D (1978) [Results in pulmonary tuberculosis patients under various treatment program conditions]. Bull Int Union Tuberc 53: 70-75.

18. Dye C, Garnett GP, Sleeman K, Williams BG (1998) Prospects for worldwide tuberculosis control under the WHO DOTS strategy. Directly observed short-course therapy. Lancet 352: 1886-1891.

19. Wong EB, Omar T, Setlhako GJ, Osih R, Feldman C, et al. (2012) Causes of death on antiretroviral therapy: a post-mortem study from South Africa. PLoS One 7: e47542.

20. Etard JF, Ndiaye I, Thierry-Mieg M, Gueye NF, Gueye PM, et al. (2006) Mortality and causes of death in adults receiving highly active antiretroviral therapy in Senegal: a 7-year cohort study. AIDS 20: 1181-1189.

21. Jenkins HE, Zignol M, Cohen T (2011) Quantifying the burden and trends of isoniazid resistant tuberculosis, 1994-2009. PLoS One 6: e22927.

22. Revised National Tuberculosis Control Program I (2011) Performance data on Tuberculosis Control.

23. Boehme CC, Nicol MP, Nabeta P, Michael JS, Gotuzzo E, et al. (2011) Feasibility, diagnostic accuracy, and effectiveness of decentralised use of the Xpert MTB/RIF test for diagnosis of tuberculosis and multidrug resistance: a multicentre implementation study. Lancet 377: 1495-1505.

24. Chang K, Lu W, Wang J, Zhang K, Jia S, et al. (2012) Rapid and effective diagnosis of tuberculosis and rifampicin resistance with Xpert MTB/RIF assay: A meta-analysis. J Infect 64: 580-588.

25. Ling DI, Zwerling AA, Pai M (2008) GenoType MTBDR assays for the diagnosis of multidrug-resistant tuberculosis: a meta-analysis. Eur Respir J 32: 1165-1174.

26. Kapoor SK, Raman AV, Sachdeva KS, Satyanarayana S (2012) How did the TB patients reach DOTS services in Delhi? A study of patient treatment seeking behavior. PLoS One 7: e42458.

27. Storla DG, Yimer S, Bjune GA (2008) A systematic review of delay in the diagnosis and treatment of tuberculosis. BMC Public Health 8: 15.

28. Corbett EL, Watt CJ, Walker N, Maher D, Williams BG, et al. (2003) The growing burden of tuberculosis: global trends and interactions with the HIV epidemic. Arch Intern Med 163: 1009-1021.

29. (1984) A controlled trial of 6 months' chemotherapy in pulmonary tuberculosis. Final report: results during the 36 months after the end of chemotherapy and beyond. British Thoracic Society. Br J Dis Chest 78: 330-336.

30. Lew W, Pai M, Oxlade O, Martin D, Menzies D (2008) Initial drug resistance and tuberculosis treatment outcomes: systematic review and meta-analysis. Ann Intern Med 149: 123-134.

31. Menzies D, Benedetti A, Paydar A, Martin I, Royce S, et al. (2009) Effect of duration and intermittency of rifampin on tuberculosis treatment outcomes: a systematic review and meta-analysis. PLoS Med 6: e1000146.

32. Hong Kong Chest-British Medical Research Council (1981) Controlled trial of four twice-weekly regimens and a daily regimen all given for 6 months for pulmonary tuberculosis. Lancet: 171–174.

33. Espinal MA, Kim SJ, Suarez PG, Kam KM, Khomenko AG, et al. (2000) Standard short-course chemotherapy for drug-resistant tuberculosis: treatment outcomes in 6 countries. JAMA 283: 2537-2545.

34. Seung KJ, Gelmanova IE, Peremitin GG, Golubchikova VT, Pavlova VE, et al. (2004) The effect of initial drug resistance on treatment response and acquired drug resistance during standardized short-course chemotherapy for tuberculosis. Clin Infect Dis 39: 1321-1328.

35. Migliori GB, Espinal M, Danilova ID, Punga VV, Grzemska M, et al. (2002) Frequency of recurrence among MDR-tB cases 'successfully' treated with standardised short-course chemotherapy. Int J Tuberc Lung Dis 6: 858-864.

36. Jones-Lopez EC, Ayakaka I, Levin J, Reilly N, Mumbowa F, et al. (2011) Effectiveness of the standard WHO recommended retreatment regimen (category II) for tuberculosis in Kampala, Uganda: a prospective cohort study. PLoS Med 8: e1000427.

37. (1999) Primary multidrug-resistant tuberculosis--Ivanovo Oblast, Russia, 1999. MMWR Morb Mortal Wkly Rep 48: 661-664.

38. Menzies D, Benedetti A, Paydar A, Royce S, Madhukar P, et al. (2009) Standardized treatment of active tuberculosis in patients with previous treatment and/or with mono-resistance to isoniazid: a systematic review and meta-analysis. PLoS Med 6: e1000150.

39. Tahaoglu K, Torun T, Sevim T, Atac G, Kir A, et al. (2001) The treatment of multidrug-resistant tuberculosis in Turkey. N Engl J Med 345: 170-174.

40. Yew WW, Chan CK, Chau CH, Tam CM, Leung CC, et al. (2000) Outcomes of patients with multidrug-resistant pulmonary tuberculosis treated with ofloxacin/levofloxacin-containing regimens. Chest 117: 744-751.

41. Mitnick C, Bayona J, Palacios E, Shin S, Furin J, et al. (2003) Community-based therapy for multidrug-resistant tuberculosis in Lima, Peru. N Engl J Med 348: 119-128.

42. Orenstein EW, Basu S, Shah NS, Andrews JR, Friedland GH, et al. (2009) Treatment outcomes among patients with multidrug-resistant tuberculosis: systematic review and meta-analysis. Lancet Infect Dis 9: 153-161.

43. Cavanaugh JS, Kazennyy BY, Nguyen ML, Kiryanova EV, Vitek E, et al. (2012) Outcomes and follow-up of patients treated for multidrug-resistant tuberculosis in Orel, Russia, 2002-2005. Int J Tuberc Lung Dis 16: 1069-1074.

44. Lee J, Lim HJ, Cho YJ, Park YS, Lee SM, et al. (2011) Recurrence after successful treatment among patients with multidrug-resistant tuberculosis. Int J Tuberc Lung Dis 15: 1331-1333.

45. Franke MF, Appleton SC, Mitnick CD, Furin JJ, Bayona J, et al. (2013) Aggressive Regimens for Multidrug-Resistant Tuberculosis Reduce Recurrence. Clin Infect Dis.

46. Jacobson KR, Theron D, Victor TC, Streicher EM, Warren RM, et al. (2011) Treatment outcomes of isoniazid-resistant tuberculosis patients, Western Cape Province, South Africa. Clin Infect Dis 53: 369-372.

47. Bang D, Andersen PH, Andersen AB, Thomsen VO (2010) Isoniazid-resistant tuberculosis in Denmark: mutations, transmission and treatment outcome. J Infect 60: 452-457.

48. Cattamanchi A, Dantes RB, Metcalfe JZ, Jarlsberg LG, Grinsdale J, et al. (2009) Clinical characteristics and treatment outcomes of patients with isoniazid-monoresistant tuberculosis. Clin Infect Dis 48: 179-185.

49. Sonnenberg P, Murray J, Shearer S, Glynn JR, Kambashi B, et al. (2000) Tuberculosis treatment failure and drug resistance--same strain or reinfection? Trans R Soc Trop Med Hyg 94: 603-607.

50. Joint United Nations Programme on HIV/AIDS (UNAIDS) (2012) Global report: UNAIDS report on the global AIDS epidemic 2012. Geneva.

51. Heym B, Stavropoulos E, Honore N, Domenech P, Saint-Joanis B, et al. (1997) Effects of overexpression of the alkyl hydroperoxide reductase AhpC on the virulence and isoniazid resistance of Mycobacterium tuberculosis. Infect Immun 65: 1395-1401.

52. Enarson D, Rouillon A (1994) The epidemiological basis of tuberculosis control; Davies P, editor. London, UK: Chapman and Hall.

53. Boehme CC, Nabeta P, Hillemann D, Nicol MP, Shenai S, et al. (2010) Rapid molecular detection of tuberculosis and rifampin resistance. N Engl J Med 363: 1005-1015.

54. Richter E, Rusch-Gerdes S, Hillemann D (2009) Drug-susceptibility testing in TB: current status and future prospects. Expert Rev Respir Med 3: 497-510.
